# Berberine and Its Main Metabolite Berberrubine Inhibit Platelet Activation Through Suppressing the Class I PI3Kβ/Rasa3/Rap1 Pathway

**DOI:** 10.3389/fphar.2021.734603

**Published:** 2021-10-08

**Authors:** Can Wang, Yangyang Cheng, Yuanhui Zhang, Hongtao Jin, Zengyan Zuo, Aiping Wang, Jianmei Huang, Jiandong Jiang, Weijia Kong

**Affiliations:** ^1^ School of Chinese Materia Medica, Beijing University of Chinese Medicine, Beijing, China; ^2^ State Key Laboratory of Bioactive Substance and Function of Natural Medicines, Institute of Materia Medica, Chinese Academy of Medical Sciences and Peking Union Medical College, Beijing, China; ^3^ Department of Virology and NHC Key Laboratory of Biotechnology of Antibiotics, Institute of Medicinal Biotechnology, Chinese Academy of Medical Sciences and Peking Union Medical College, Beijing, China; ^4^ New Drug Safety Evaluation Center, Institute of Materia Medica, Chinese Academy of Medical Sciences and Peking Union Medical College, Beijing, China

**Keywords:** berberine, berberrubine, platelet activation, class I PI3Kβ, thrombus formation

## Abstract

**Background:** Berberine (BBR), a natural product, was reported to inhibit platelet aggregation; however, the molecular mechanisms remain unclear. This study aims to investigate the effects and mechanisms of BBR in inhibiting platelet activation and thrombus formation.

**Methods:** Flow cytometry, immunofluorescence, and Western blot were used to determine the inhibitory effects and mechanisms of BBR and its main metabolite berberrubine (M2) on platelet activation *in vitro* and *ex vivo*. Purified integrin αIIbβ3, class I PI3K kit, and molecular docking were used to identify the possible targets of BBR and M2. A carrageenan-induced mouse thrombosis model was used to evaluate the effects of BBR on thrombus formation *in vivo*.

**Results:** *In vitro*, BBR and M2 significantly inhibited ADP-induced integrin αIIbβ3 activation, reduced the level of P-selectin on the platelet membrane, and suppressed the binding of fibrinogen to the platelets. In this process, BBR and M2 greatly suppressed the PI3K/Akt pathway and inhibited Rasa3 membrane translocation and Rap1 activation. Furthermore, BBR and M2 selectively inhibited class I PI3Kβ, perhaps through binding to its active site. The activities of BBR were stronger than those of M2. After oral administration, BBR significantly inhibited the PI3K/Akt pathway and Rap1 activation and suppressed ADP-induced platelet activation and carrageenan-induced thrombosis in mice without prolonging bleeding time.

**Conclusions:** We reveal for the first time the possible targets and mechanisms of BBR and M2 in inhibiting platelet activation. Our research may support the future clinical application of BBR as an antiplatelet drug in the prevention or treatment of thrombotic diseases.

## Introduction

Thrombotic diseases and the related cardiovascular or cerebrovascular events, such as myocardial infarction or stroke, are the leading causes of mortality and morbidity worldwide (Caron and Anand, 2017; Thomas et al., 2018; Wang et al., 2018). Inhibiting thrombus formation is a directly effective way for the prevention or treatment of cardiovascular or cerebrovascular diseases (Asada et al., 2018). Currently, there are two main types of drugs used in clinics for the treatment of thrombus formation: anticoagulant drugs (Mega and Simon, 2015; Honda et al., 2016) and antiplatelet drugs (Jing et al., 2011; Patrono et al., 2017; Binsaleh et al., 2018). However, bleeding risk is a common challenge for these drugs in clinical use (Patrono et al., 2017). Moreover, a recent study showed that warfarin, a kind of vitamin K antagonist that has been used for prophylaxis or the treatment of thromboembolic events for 64 years, may increase the risk of myelodysplastic syndrome (Verma et al., 2019). Therefore, the development of more safe and effective antithrombotic drugs is of scientific and clinical significance.

A variety of natural products isolated from traditional Chinese medicine have shown good safety and pharmacological activity in anti-thrombosis (Huang et al., 2010; Song et al., 2019). Berberine (BBR), a natural product isolated from *Coptis chinensis*, has been used for the treatment of bacterial diarrhea in clinics for many years in China (National Pharmacopoeia Committee, 2015), and no side effects of hemorrhagic tendencies have been reported (Wang et al., 2018). BBR has beneficial effects against a variety of chronic diseases (Kong et al., 2004; Zhang et al., 2010; Pirillo and Catapano, 2015; Zou et al., 2017). As early as the 1980s, researchers had reported that BBR was able to inhibit platelet aggregation, both in preclinical and clinical studies (Chen and Xie, 1986; Chu et al., 1989; Huang et al., 1989). BBR also inhibited thrombus formation effectively in animal models. For example, we previously reported that BBR significantly inhibited thrombus formation in the inferior vena cava in rats fed a normal or high-fat diet (Wang et al., 2018).

**FIGURE 1:**
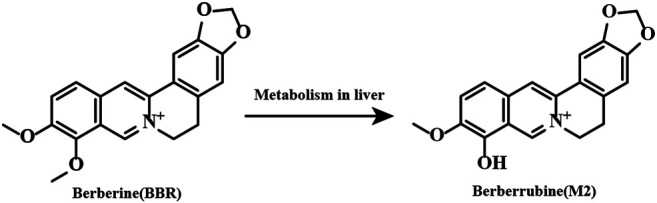
Chemical structures of BBR and its main metabolite M2.

Currently, the mechanisms of BBR in inhibiting platelet activation and aggregation remain unclear. In addition, whether or not the metabolites of BBR have antiplatelet activities is unknown. Among the BBR metabolites, berberrubine (M2) (Figure 1) is the main metabolite and accounts for 65.1% of all BBR metabolites in the liver (Tan et al., 2013). Therefore, in this research, we aim to investigate the antiplatelet effects and possible mechanisms of BBR and M2, and our results prove that BBR and M2 suppress platelet activation through inhibiting the class I PI3Kβ/Rasa3/Rap1 pathway, which is related to the antithrombotic effect of BBR.

## Materials and Methods

### Mice

All C57BL/6N mice (males, 6–7 weeks old, 18–20 g) and BALB/c mice (males, 6–7 weeks old, 18–20 g) were purchased from Beijing Vital River Laboratory Animal Technology Co., Ltd. The mice were kept in a room at a temperature of 22–24°C and humidity of 45% with a 12-h day and night cycle (lighting time 8:00–20:00). All animal experiments were reviewed and approved by the Ethics Committee of the Institute of Materia Medica, Chinese Academy of Medical Sciences (CAMS) and Peking Union Medical College (PUMC) (No. 00005787, No. 00005788, No. 00005789). After the experiments, all mice were anesthetized with 2% isoflurane inhalation and sacrificed with cervical dislocation to perform animal euthanasia. To protect animals used for scientific purposes, all animal procedures which were performed conformed to the guidelines from EU Directive 2010/63/EU for animal experiments.

### Reagents and Kits

Sodium citrate tribasic dihydrate, prostaglandin E1 (PGE1), N-[2-Hydroxyethyl] piperazine-N'-[2-ethanesulfonic acid (HEPES), berberine chloride, ADP, 3,3′,5,5′-tetramethylbenzidine (TMB) liquid substrate (#T4444), aspirin (Asp), and a stop reagent for the TMB substrate (#S5814) were obtained from Sigma-Aldrich (St. Louis, MO, United States). Berberrubine chloride (M2) was obtained from Chengdu Herbpurify CO., LTD. (Chengdu, Sichuan, China). PE-labeled rat anti-mouse JON/A monoclonal antibody (#M023-2), PE-labeled rat IgG polyclonal antibody (#P190-2), FITC-labeled rat anti-mouse p-selectin (CD62P) monoclonal antibody (#M130-1), and FITC-labeled rat IgG polyclonal antibody (#P190-1) were purchased from Emfret Analytics (Würzburg, Germany). Eight-well chambered glass coverslips (#155411PK), FluoroNunc 96-well plates, Calcein-AM (#C3099), TRITC–phalloidin (#R415), the M-PER™ Mammalian Protein Extraction Reagent (#78501), the Mem-PER™ Plus Membrane Protein Extraction Kit (#89842), and the active Rap1 pull-down and detection kit (#16120) were purchased from Thermo Fisher Scientific Inc. (Waltham, MA, United States). Mouse fibrinogen (#CT15) was obtained from Oxford Biomedical Research (Oxford, United Kingdom). Human GPIIbIIIa (#GP2b3a) was obtained from Enzyme Research Laboratories (South Bend, IN, United States). Fibrinogen plasminogen-depleted (#341578) and rabbit anti-FcRγ polyclonal antibodies (#06–727) were purchased from Millipore (Billerica, MA, United States). Fibrinogen antibody (HRP) (#60R-1012) was purchased from Fitzgerald (Acton, MA, United States). Tirofiban hydrochloride monohydrate (#HY-17369) and clopidogrel (Clop) hydrogen sulfate were obtained from MedChemExpress (Shanghai, China). Rabbit anti-p-Akt (Ser^473^) (#4058), rabbit anti-Akt (#9272), rabbit anti-ERK (#9102) antibodies, and wortmannin (Wtm) were sourced from Cell Signaling Technology, Inc. (Danvers, MA, United States). Mouse anti-Rasa3 monoclonal antibody (#SC-398283) and integrin β3 antibody (B-7) (#SC-46655) were sourced from Santa Cruz Biotechnology, Inc. (Dallas, TX, United States). ADP-Glo lipid kinase systems (#V1691) were purchased from Promega Corporation (Madison, WI, United States). TGX221 was purchased from Cayman Chemical Company (Ann Arbor, MI, United States).

### Preparation of Mouse Washed Platelets

Mouse washed platelets (WPs) were prepared as described previously (Blue et al., 2008). Briefly, mice were anaesthetized with 2% isoflurane inhalation, and retro-orbital blood samples were collected into the sodium citrate solution (blood volume: sodium citrate solution, 9:1). The anticoagulated whole blood sample was centrifuged at 22°C at 650 *g* for 4 min to obtain platelet-rich plasma (PRP). Then, PGE1 (final concentration 1 μM) was added to PRP and mixed well, and the PRP was centrifuged at 22°C at 1,200 *g* for 8 min to obtain the precipitation of WPs. Then, the WPs were resuspended in HEPES-modified Tyrode buffer (HBMT; 10 mM HEPES, 138 mM NaCl, 12 mM NaHCO_3_, 2.7 mM KCl, 0.4 mM NaH_2_PO_4_, 0.1% glucose, 0.35% BSA, 2 mM CaCl_2_, and 1 mM MgCl_2_, pH = 7.4). The platelet concentration was adjusted to 3 × 10^8^/ml by counting with a hemocytometer.

### Flow Cytometry

The methods of flow cytometry were performed as described previously (Deng et al., 2016). Briefly, the suspension of the WPs was divided into several groups and treated with the vehicle control (0.1% DMSO) or different compounds at 37°C for 10 min and then stimulated with 10 μM of ADP for 10 min. After treatment, the WPs were fixed with 1% paraformaldehyde at room temperature for 15 min. The platelet suspension was centrifuged at 22°C at 1,200 *g* for 8 min, and then the precipitation was resuspended in HBMT. Each group was divided into four parts, and they were incubated with the PE-conjugated JON/A antibody (selectively binding to the high-affinity conformation of mouse integrin αIIbβ3), FITC-labeled rat anti-mouse P-selectin monoclonal antibody, or the negative control IgGs for 15 min. After terminating the reaction with 400 μl PBS, the platelets were analyzed using a BD FACSCalibur flow cytometer (BD-Biosciences, Heidelberg, Germany) or BD Accuri C6 flow cytometer (BD-Biosciences, Heidelberg, Germany). All data were analyzed using FlowJo software (BD-Biosciences, Heidelberg, Germany).

### Immunofluorescence

Immunofluorescence was performed as described before with slight modifications (Blue et al., 2008). Briefly, eight-chambered glass coverslips (Nunc) were coated with 200 μl fibrinogen (50 μg/ml). After incubating at 4°C overnight, each chamber was washed three times with Tris/saline (100 mM sodium chloride, 50 mM Tris/HCl, pH = 7.4). And then, each chamber was blocked with HBMT at room temperature for 1 h and washed two times with Tris/saline. After treatment, WPs were added to the eight-well chambered glass coverslips coated with fibrinogen and incubated at room temperature for 1 h, then each chamber was washed with HBMT (containing 2 mM CaCl_2_ and 1 mM MgCl_2_) four times. The adherent platelets were fixed with 1% paraformaldehyde at room temperature for 15 min and washed with PBS three times. Then, the platelets were treated with 0.1% Triton X-100 for 15 min and washed with PBS for another three times. After being blocked with 1% BSA/PBS at room temperature for 1 h, the adherent platelets were incubated with the TRITC–phalloidin working solution at room temperature for 1 h and washed with PBS again three times. Finally, immunofluorescence images were taken using an Olympus IX71 fluorescent inverted microscope with a 40× objective and 10× eyepiece.

### Platelet Adhesion Assay

Platelet adhesion to fibrinogen was performed as described before with slight modifications (Blue et al., 2008; Su et al., 2016). Briefly, human fibrinogen was diluted and dissolved into the fibrinogen solution with a final concentration of 50 g/ml. 200 μl of the fibrinogen solution (50 μg/ml) was added to FluoroNunc 96-well plates. After incubating at 4°C overnight, the plates were washed with 200 μl of Tris/saline three times. Then, the wells were blocked with 200 μl of HBMT containing 2% BSA for 1 h at room temperature and washed with Tris/saline two times. During the period, WPs were treated with different concentrations of BBR or M2 or the vehicle control (0.1% DMSO) at 37°C for 10 min and then stimulated with 10 μM ADP for 10 min. After treatment, WPs were labeled with calcein-AM at a final concentration of 16 μM at room temperature for 30 min in the absence of light. And then, 100 μl labeled platelets were added into coated FluoroNunc 96-well plates and incubated at room temperature for 1 h. After washing four times with 200 μl HBMT containing 2 mM CaCl_2_ and 1 mM MgCl_2_, 100 μl HBMT containing 2 mM CaCl_2_ and 1 mM MgCl_2_ was added. The fluorescence intensity was measured using the EnSpire Multimode Plate Reader (PerkinElmer, Waltham, MA, United States) to represent the relative number of adherent platelets (excitation wavelengths/emission wavelengths: 490 nm/515 nm).

### Protein Extraction and Western Blot

After treatment, the WPs were centrifuged at 1,200 *g* at 22°C for 8 min to obtain the precipitation. The M-PER™ Mammalian Protein Extraction Reagent was added to obtain whole cell lysis, and the Mem-PER™ Plus Membrane Protein Extraction Kit was used to obtain membrane proteins and cytosolic proteins following the kit's instructions. Western blot was performed as described before (Wang et al., 2016). Briefly, 20 μl of the cell lysate was used for 10% sodium dodecyl sulfate polyacrylamide gel electrophoresis, and the proteins were transferred from the gels onto the PVDF membranes. After blocking, the blots of target proteins were detected with specific primary antibodies and appropriate secondary antibodies. The signals were developed using the ECL kit (EMD Millipore Corporation). After scanning and quantification, the levels of p-AKT (Ser^473^) were normalized to those of AKT and plotted as indicated. The ratios of the Rasa3 protein in the membrane to FcRγ and those in the cytoplasm to ERK were calculated. The original scans of Western blot are shown in Supplementary Figures S1–4.

### Rap1 Activation Assay

The Rap1 activation assay was performed following the kit's instructions; GTPγS (positive control) and GDP (negative control) were used to ensure that the pull-down procedures worked properly. Briefly, after treatment, the WPs precipitation was prepared and split with 300 μl lysis/binding/wash buffer on ice for 5 min. The supernatant was collected by centrifuging the lysate at 4°C for 15 min and added to the spin cup containing the glutathione resin and GST-RalGDS-RBD. The reaction mixture was vortexed and incubated at 4°C for 1 h with gentle rocking. The spin cups were centrifuged with collection tubes at 6,000 *g* for 1 min, and the resin was washed three times. After adding 50 μl 2× reducing sample buffer (1 part β-mercaptoethanol to 20 parts 2× SDS sample buffer), the samples were vortexed and incubated at room temperature for 2 min and then centrifuged at 6,000 *g* for 2 min. The spin cup containing the resin was removed and discarded. The eluted samples were heated for 5 min at 95–100°C. Western blot was used to detect the pull-down of GTP-Rap1 (Rap1 active form). The original scans of Western blot are shown in Supplementary Figures S1, 3.

### Fibrinogen Binding to Purified Integrin αIIbβ3

The purified integrin αIIbβ3 binding assay was performed as described previously (Blue et al., 2008; Su et al., 2016). Briefly, 96-well plates were coated with the integrin β3 antibody (B-7) (10 μg/ml) at 4°C overnight and, then, were blocked with 3.5% BSA at room temperature for 1 h. Purified integrin αIIbβ3 was diluted in buffer A (50 mM Tris/HCl, 100 mM NaCl, 1 mM CaCl_2_, 1 mM MgCl_2_, 1% BSA, and 0.0035% Triton X-100) to 10 μg/ml, added to the wells, and captured by the integrin β3 antibody (B-7) for 2 h at 37°C. The wells were then washed three times with buffer A. Fibrinogen, prepared in buffer A (20 μg/ml), was incubated in the well plates for 2 h at 37°C with or without the studied compounds or control solutions. The wells were washed three times with buffer A and then incubated with a horseradish peroxidase (HRP)–conjugated polyclonal anti-fibrinogen antibody (1:1,000 in buffer A) for 1 h at room temperature. For color development, the wells were washed three times. 100 μl of a peroxidase substrate (TMB) was added, and the reaction was terminated after 30 min by adding 100 μl of the stop reagent for the TMB substrate. Finally, the absorbance value was determined by spectrophotometry at 450 nm.

### Class I PI3K Activity Assays

The effects of BBR and M2 on different isoforms of class I PI3K were determined following the kit's instructions. Briefly, different isoforms of recombinant class I PI3K enzymes were mixed with the lipid kinase substrate which contained 1 mg of phosphoinositol-4, 5-bisphosphate (PIP2) and 3 mg of phosphatidylserine. Together with the studied compounds, the reactions were incubated at room temperature for 20 min. After incubation, the ATP solution (25 μM) was added to each well. The assay plate was covered and incubated at room temperature for 1 h. The ADP-Glo reagent containing 10 mM of MgCl_2_ was used to stop the reaction and deplete the unconsumed ATP. Then, the kinase detection reagent was added to each well and incubated at room temperature for 40 min to convert ADP to ATP. The amount of newly synthesized ATP was detected with a coupled luciferin/luciferase reaction and used to represent class I PI3K activity.

For the ATP competition experiments, different isoforms of class I PI3K were incubated with the substrate and 1 μM of BBR or M2 for 20 min, and lipid kinase reactions were performed in the presence of different concentrations of ATP (6.25–400 μM).

### Molecular Docking

The 2D structures of BBR and M2 were drawn using ChemBioDraw 2014 and converted to 3D structures in MOE through energy minimization. The 3D structure of the mouse protein PI3K p110β was downloaded from the RCSB Protein Data Bank (PDB ID was 2Y3A). Prior to docking, the force field of AMBER10:EHT and the implicit solvation model of the reaction field (R-field) were selected. MOE-Dock was used for molecular docking simulations of molecules with proteins. The docking workflow followed the “induced fit” protocol, in which the side chains of the receptor pocket were allowed to move according to ligand conformations, with a constraint on their positions. The weight used for tethering side chain atoms to their original positions was 10. For each ligand, all docked poses were ranked by London dG scoring first, then a force field refinement was carried out on the top 20 poses followed by the rescoring of GBVI/WSA dG. The conformations with the lowest free energies of binding were selected as the best (probable) binding modes. Molecular graphics were generated by PyMOL.

### Determination of *Ex Vivo* Platelet Activation and Signaling Pathways

C57BL/6N mice were randomly divided into five groups (n = 5 each), and each group received the following treatment for 14 days: vehicle group—intragastric (i.g.) administration of normal saline (NS, 0.1 ml/10 g), and the WPs were incubated with the vehicle control (0.1% DMSO); vehicle + ADP group—i.g., administration of NS, and the WPs were stimulated with ADP (10 μM); BBR100 + ADP group—i.g., administration of BBR (100 mg/kg), and the WPs were stimulated with ADP; BBR200 + ADP group—i.g., administration of BBR (200 mg/kg), and the WPs were stimulated with ADP; Clop10 + ADP group—i.g., administration of Clop (10 mg/kg) and the WPs were stimulated with ADP.

After treatment, the PE-conjugated JON/A antibody and FITC-labeled P-selectin antibody binding of the platelets were determined by flow cytometry. The proteins were extracted, and p-Akt and GTP-Rap1 levels were determined by the pull-down assay and Western blot as described above.

### Tail Bleeding Assay

The mice tail bleeding time was determined according to a method described previously with modifications (Song et al., 2019). Briefly, 30 C57BL/6N mice were randomly divided into six groups (n = 5 each). Before drug administration, the mice were anaesthetized with 2% isoflurane inhalation, and then the tail bleeding time was determined. A 5-mm tail tip of each mouse was cut off using a surgical blade, and the transected tail tip was transferred onto a clean filter paper. The tail bleeding time was recorded every 30 s s until the bleeding completely stopped.

Twenty-five hours before the second measurement, the mice were orally administrated with BBR (200 mg/kg), Clop (10 mg/kg), Asp (100 mg/kg), BBR (200 mg/kg) + Clop (10 mg/kg), BBR (200 mg/kg) + Asp (100 mg/kg), or vehicle (1% carboxymethyl cellulose sodium). All mice were given the same volume at 0.1 ml/10 g. One hour before the second measurement, all groups' mice were given the studied compounds or the vehicle control once again in the same way as described above. One hour after the second dose administration, the mice were anaesthetized with 2% isoflurane inhalation, and then the tail bleeding time was measured again.

### Carrageenan-Induced Thrombus Formation

The methods of carrageenan-induced thrombus formation in mice were performed as described previously with modifications (Li et al., 2019). Forty BALB/c mice were randomly divided into the following six groups: vehicle group (n = 5), which was i.g. administered with NS (0.1ml/10 g), vehicle + carrageenan group (n = 7), BBR 50 mg/kg + carrageenan group (n = 7), BBR 100 mg/kg + carrageenan group (n = 7), BBR 200 mg/kg + carrageenan group (n = 7), and Clop 10 mg/kg + carrageenan group (n = 7).

The treatment lasted for 12 days. On day 12, the mice in the vehicle control group were intraperitoneally (i.p.) injected with NS (50μl/10 g), while the mice in the other groups were i.p. injected with 0.5% carrageenan solution (50 μl/10 g, final dose 50 mg/kg). Two days after carrageenan injection, the mice were anaesthetized with 2% isoflurane inhalation. The tails of mice were photographed quickly; the original images of mice tail thrombosis are shown in Supplementary Figure S4, and both the length of thrombus in and the full length of the tails of the mice were measured using a steel ruler. The thrombosis rate of the tails of the mice was calculated by the following formula: thrombus length/whole tail length × 100.

After photography and measurement, the blood samples were collected through retro-orbital puncture, and the tails of the mice were harvested and fixed in 4% paraformaldehyde. For the tails of the mice, paraffin sections were prepared at 2, 4, and 6 cm away from the tail tips for hematoxylin and eosin (H&E) staining. The thrombus areas were calculated using Image-pro plus 6.0 (Media Cybernetics, Inc., Rockville, MD, United States) and presented as percentages of the whole mice tail vessels. The WPs were prepared, and the proteins were extracted for Western blot to detect the p-AKT/AKT level. The original scans of Western blot are shown in Supplementary Figure S4.

### Statistical Analysis

For *in vitro* experiments, the values are expressed as mean ± standard deviation (SD) of three to five repeated experiments. For *ex vivo* and *in vivo* experiments, the values are expressed as mean ± SD of five or seven mice in each group. After validation of the test for homogeneity of variance, one-way ANOVA followed by the Newman–Keuls test for multiple comparisons was used to analyze significant differences among multiple studying groups. In all experiments, *p* < 0.05 was considered to be statistically significant.

## Results

### BBR and M2 Inhibit Platelet Activation Induced by ADP *in vitro*

**FIGURE 2:**
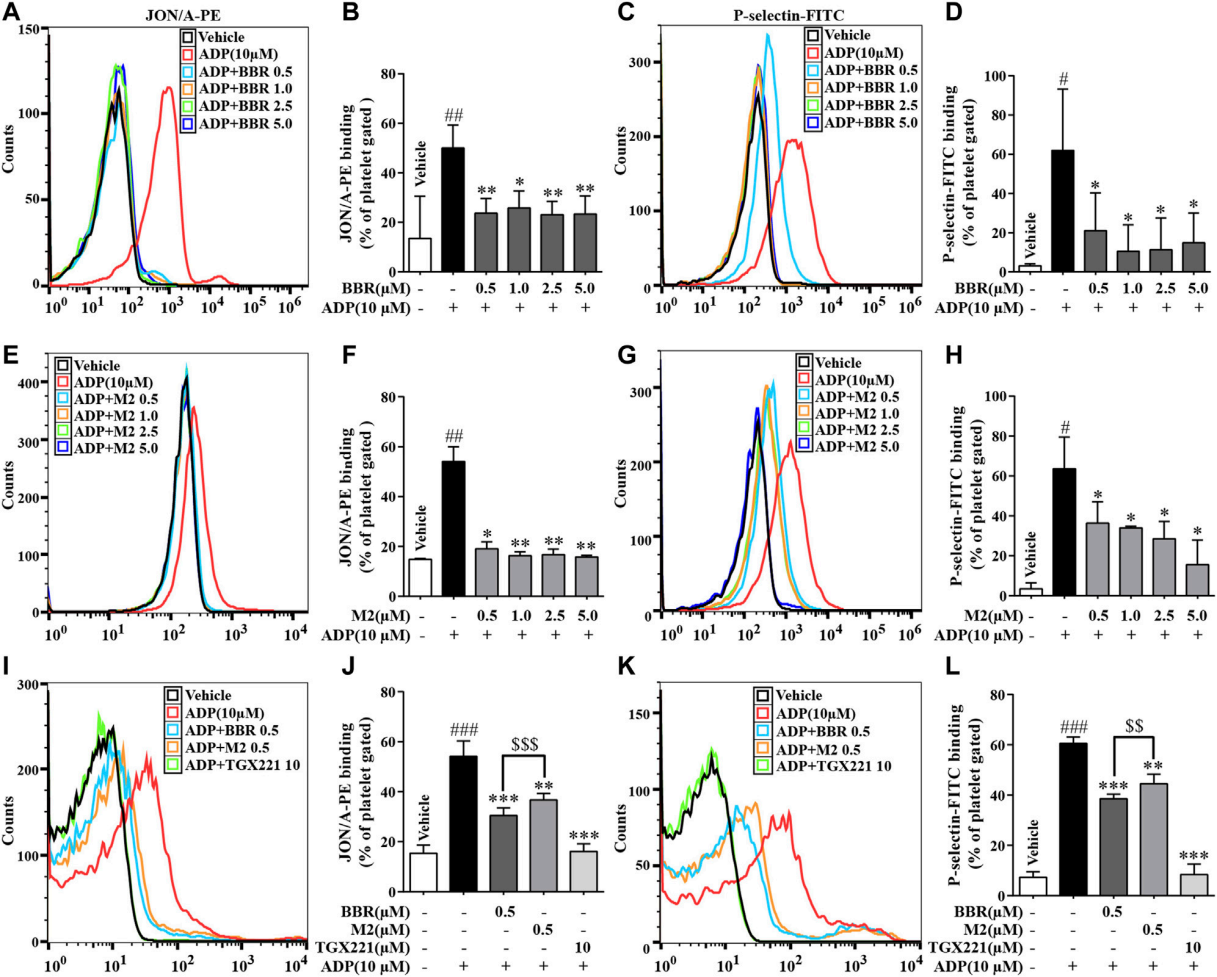
Effects of BBR and M2 on platelet activation induced by ADP *in vitro*. The WPs were pretreated with different compounds or the vehicle control (0.1% DMSO) for 10 min and stimulated with 10 μM of ADP for 10 min. After treatment, the PE-conjugated JON/A antibody and the FITC-conjugated P-selectin antibody were used to detect the integrin αIIbβ3 active form and P-selectin expression on the platelet surface by flow cytometry. The positive platelet ratios in gated platelets were calculated and plotted as indicated. **(A-D)** The WPs were pretreated with BBR at indicated concentrations. **(E-H)** The WPs were pretreated with M2 at indicated concentrations. **(I-L)** The WPs were pretreated with BBR, M2, or TGX221 at indicated concentrations. The histograms **(A, C, E, G, I, K)** are representatives of four or five independent flow cytometry experiments, and the quantitative results **(B, D, F, H, J, L)** are expressed as their mean ± SD. Statistically significant differences compared with the vehicle control group are indicated by ^#^ *p* < 0.05, ^##^ *p* < 0.01, and ^###^ *p* < 0.001. Statistically significant differences compared with the ADP group are indicated by *p < 0.05, **p < 0.01, and ***p < 0.001, and statistically significant differences between the BBR and M2 groups are indicated by ^$$^ *p* < 0.01, ^$$$^ *p* < 0.001.

As shown in Figure 2, compared with the vehicle control group (*p* < 0.05, *p* < 0.01, or *p* < 0.001), the percentages of positive platelets with PE-conjugated JON/A antibody binding or FITC-conjugated P-selectin antibody binding on the surface increased significantly when platelets were stimulated by ADP. Compared with the ADP-treated platelets (*p* < 0.05 or *p* < 0.01), pretreatment with different concentrations of BBR (0.5–5.0 μM) (Figures 2A–D) or M2 (0.5–5.0 μM) (Figures 2E–H) significantly decreased the percentages of positive platelets.

The inhibitory effects of BBR and M2 on platelet activation at the same concentration were compared. Compared with the ADP group, 0.5 μM of BBR or M2 reduced the proportions of positive platelets binding to the PE-conjugated JON/A antibody by about 43.7% (*p* < 0.001) and 32.1% (*p* < 0.01), respectively (Figures 2I,J) and reduced the proportions of positive platelets binding to the FITC-conjugated P-selectin antibody by about 36.5% (*p* < 0.001) and 26.6% (*p* < 0.01), respectively (Figures 2K,L). These results indicated that both BBR and M2 could significantly inhibit platelet activation induced by ADP, and BBR has a stronger inhibitory effect on platelet activation than M2 at the same concentration (*p* < 0.001 or *p* < 0.01). As a positive control, TGX221 potently suppressed platelet activation induced by ADP, and the proportions of positive platelets almost returned to normal levels after TGX221 treatment (Figures 2I–L).

### BBR and M2 Inhibit Fibrinogen Binding to Platelets but Have no Effect on Fibrinogen Binding to Purified Integrin αIIbβ3

**FIGURE 3:**
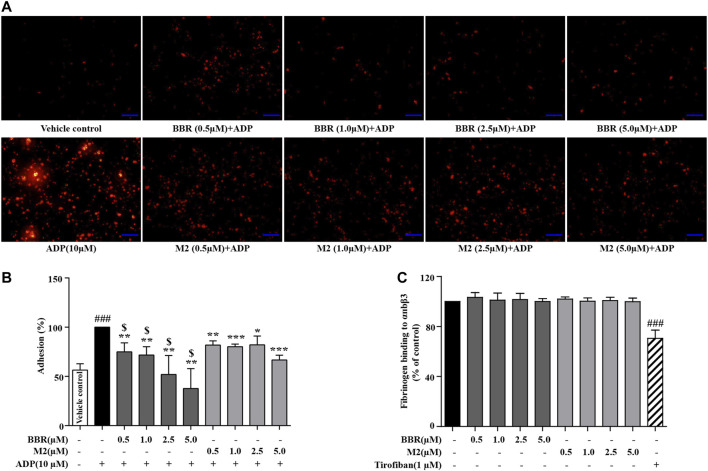
Effects of BBR and M2 on the binding of fibrinogen to the platelets and purified αIIbβ3 *in vitro*. The WPs were pretreated with the vehicle control (0.1% DMSO), BBR, or M2 at indicated concentrations for 10 min and stimulated with 10 μM of ADP for 10 min. **(A)** The platelets were incubated with fibrinogen coated in glass coverslips and stained with TRITC–phalloidin. Images were taken using a fluorescence microscope and are presented as representative of three independent experiments (× 400, scale bar = 100 µm). **(B)** The platelets were stained with calcein-AM and incubated in glass coverslips coated with fibrinogen. The fluorescence intensities were measured for the quantification of platelet adhesion, which are plotted as percentages of the ADP group. The values are expressed as mean ± SD of three independent experiments. ^###^ *p* < 0.001 vs that of the vehicle control group; *p < 0.05, **p < 0.01, and ***p < 0.001 vs that of the ADP group. ^$^ *p* < 0.05 vs that of the M2 group at the same concentration. **(C)** Fibrinogen was added to wells which were bound with purified αIIbβ3, together with the vehicle control, at different concentrations of BBR, M2, or 1 μM of tirofiban. After treatment, the residual fibrinogen was measured and plotted as percentages of the vehicle control. The values are expressed as mean ± SD of seven independent experiments. Statistically significant differences when compared with the vehicle control group are indicated by ^###^ *p* < 0.001.

As shown in Figure 3A, TRITC–phalloidin staining indicated that as compared to the vehicle control group, a large number of the WPs bound to coated fibrinogen after stimulation with ADP, as indicated by a significant increase of the red-stained area. BBR (0.5–5.0 μM) or M2 (0.5–5.0 μM) inhibited the binding of the platelets to fibrinogen, as indicated by an obvious reduction of the red-stained area after pretreatment. To quantitatively analyze the inhibitory effects of BBR and M2, the platelets were stained with calcein-AM, incubated with coated fibrinogen, and fluorescence intensities were determined. As shown in Figure 3B, as low as 0.5 μM of BBR or M2 was able to cause a significant reduction of the binding of platelets to fibrinogen (*p* < 0.01 vs ADP-induced platelets), and when the concentration of BBR or M2 reached 5.0 μM, a greater extent of binding inhibition was observed (*p* < 0.01 or *p* < 0.001 vs ADP-induced platelets). Similar to the inhibitory effects on platelet activation, BBR exhibited a more potent effect to inhibit the binding of fibrinogen to the platelets than did M2 at the same concentration (*p* < 0.05) (Figure 3B).

To explore whether or not BBR and M2 could directly inhibit the binding of integrin αIIbβ3 to fibrinogen, purified αIIbβ3 was used in our experiments. As shown in Figure 3C, neither BBR nor M2 had any influence on the binding of fibrinogen to purified integrin αIIbβ3. For comparison, tirofiban, a positive control used in the experiments, suppressed the binding of fibrinogen to purified integrin αIIbβ3 significantly (*p* < 0.001 vs vehicle control) (Figure 3C).

### BBR and M2 Suppress the PI3K/Akt Signaling Pathway and Inhibit Rap1 Activation Induced by ADP

**FIGURE 4:**
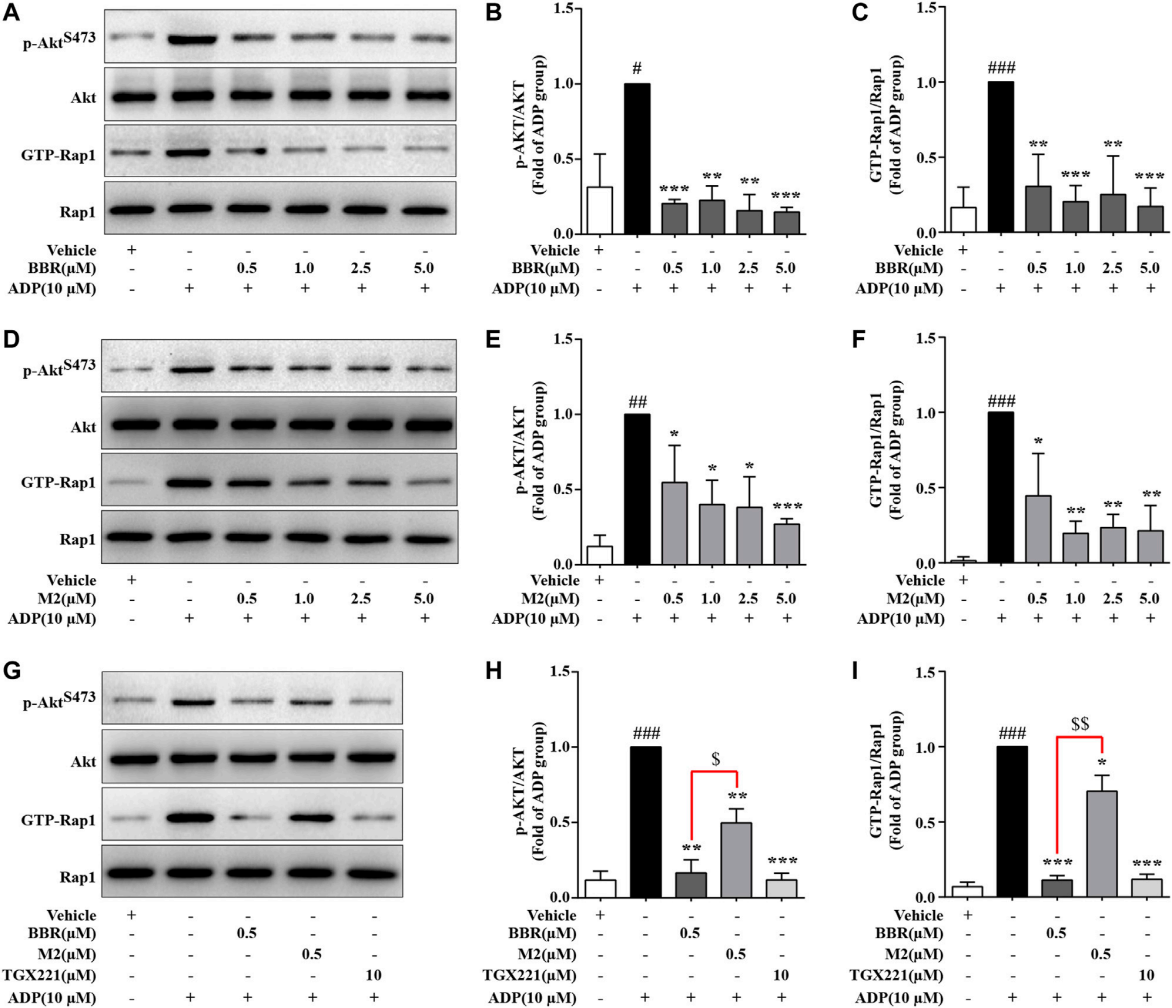
Effects of BBR and M2 on Akt phosphorylation and Rap1 activation in ADP-stimulated platelets *in vitro*. The WPs were pretreated with the studied compounds or the vehicle control (0.1% DMSO) for 10 min and stimulated with 10 μM of ADP for 10 min. After treatment, the platelets were lysed for protein extraction and Western blot used to detect the levels of p-Akt^S473^, Akt, and Rap1. In parallel experiments, platelet lysates were used to –pull down and detect GTP-Rap1. **(A-C)** The WPs were pretreated with BBR or the vehicle control as indicated. **(D-F)** The WPs were pretreated with M2 or the vehicle control as indicated. **(G-I)** The WPs were pretreated with BBR (0.5 μM), M2 (0.5 μM), TGX221 (10 μM), or the vehicle control as indicated. Representative blots are presented **(A, D, G)**. After quantification, the expression levels of p-Akt^S473^ and GTP-Rap1 were normalized to those of Akt and Rap1, respectively, and plotted as indicated **(B, C, E, F, H, I)**. Data are expressed as mean ± SD of three or four independent experiments. Statistically significant differences compared with the vehicle control group are indicated by ^#^ *p* < 0.05, ^##^ *p* < 0.01, and ^###^ *p* < 0.001. Statistically significant differences compared with the ADP group are indicated by *p < 0.05, **p < 0.01, and ***p < 0.001, and statistically significant differences between the BBR and M2 groups are indicated by ^$^ *p* < 0.05 and ^$$^ *p* < 0.01.

As the activation of PI3K/Akt and Rap1 is crucial for ADP-induced platelet activation (Zhu et al., 2017; Stefanini and Bergmeier, 2019), the effects of BBR and M2 on these molecules were determined. As shown in Figure 4, compared with the vehicle control group (*p* < 0.05, *p* < 0.01, or *p* < 0.001), the phosphorylation of Akt, which reflected the activation of PI3K, and the level of GTP-Rap1 (Rap1 active form) in the platelets simultaneously increased after induction with 10 μM of ADP. BBR and M2 had no effect on the levels of total Akt and Rap1. However, compared with the ADP treatment group, pretreatment with BBR (0.5–5.0 μM) (*p* < 0.01 or *p* < 0.001) (Figures 4A–C) or M2 (0.5–5.0 μM) (*p* < 0.05, *p* < 0.01, or *p* < 0.001) (Figures 4D–F) significantly inhibited Akt phosphorylation and reduced the GTP-Rap1 level upon ADP stimulation. Moreover, the effects of BBR were stronger than those of M2 (*p* < 0.05 or *p* < 0.01) when administered at the same concentration (Figures 4G–I). As a positive control, TGX221 had potent inhibitory effects on Akt phosphorylation and Rap1 activation stimulated by ADP (*p* < 0.001 vs ADP treatment group) (Figures 4G–I).

### BBR and M2 Inhibit Rasa3 Membrane Translocation Upon ADP Stimulation

**FIGURE 5:**
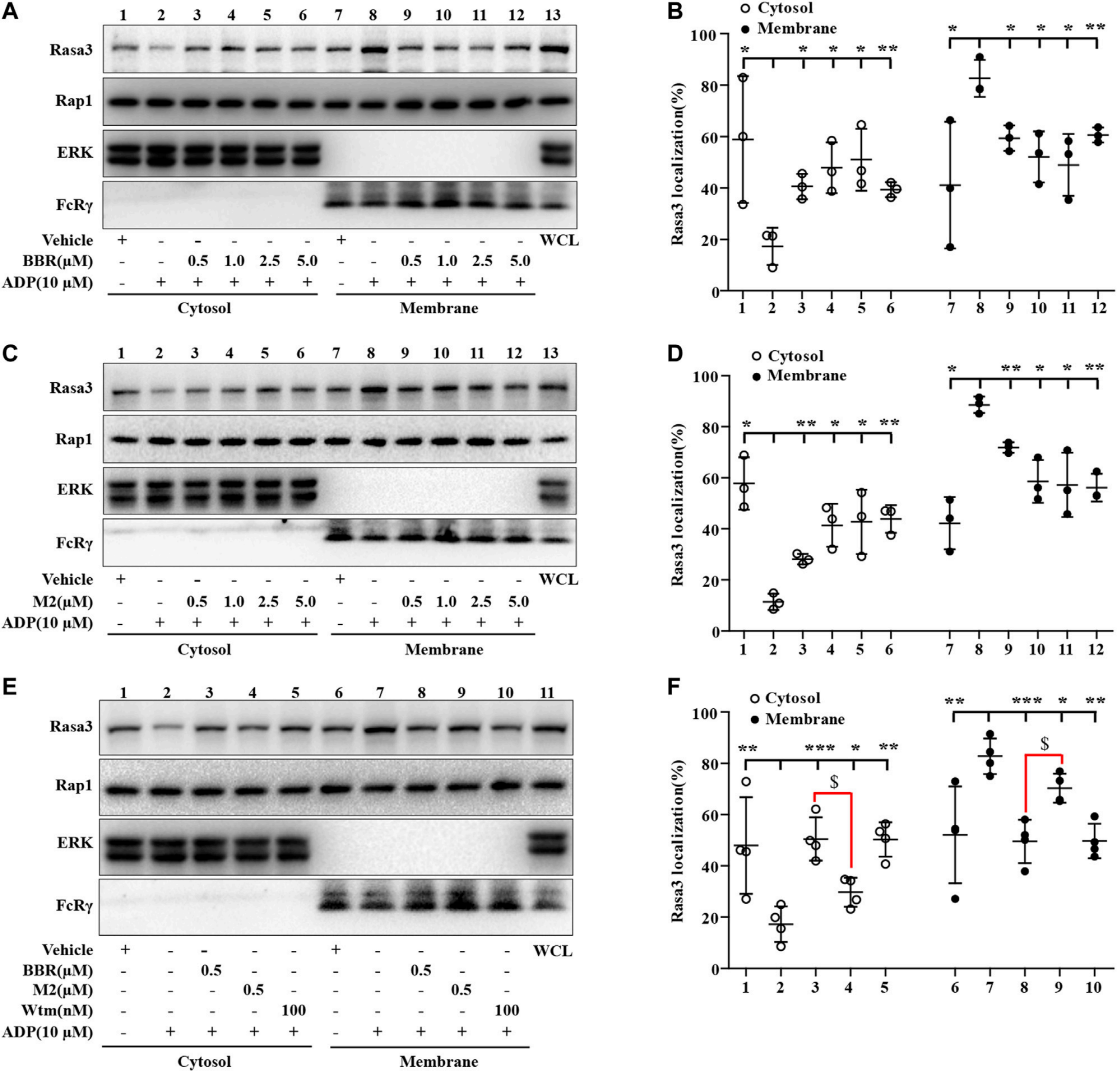
Effects of BBR and M2 on Rasa3 membrane translocation upon ADP stimulation *in vitro*. The WPs were pretreated with the studied compounds or the vehicle control (0.1% DMSO) for 10 min, and stimulated with 10 μM of ADP for 10 min. After treatment, the cytosolic and membrane proteins were extracted for Western blot to detect Rasa3, Rap1, ERK (cytosolic control protein), and FcRγ (membrane control protein). **(A-B)** The WPs were pretreated with BBR or the vehicle control as indicated. **(C-D)** The WPs were pretreated with M2 or the vehicle control as indicated. **(E-F)** The WPs were pretreated with BBR (0.5 μM), M2 (0.5 μM), Wtm (100 nM), or the vehicle control as indicated. Representative blots are presented **(A, C, E)**. After quantification, the expression levels of cytosolic and membrane Rasa3 were normalized to those of ERK and FcRγ, respectively, and plotted as indicated **(B, D, F)**. Data are mean ± SD of three or four independent experiments. Statistically significant differences compared with ADP group are indicated by *p < 0.05, **p < 0.01, ***p < 0.001, and statistically significant differences between the BBR and M2 groups are indicated by ^$^ *p* < 0.05.

As the Rap1 GTPase-activating protein (GAP) Rasa3 was involved in the modulation of Rap1 activation in the platelets (Battram et al., 2017), the effects of BBR and M2 on Rasa3 were investigated in our experiments. As shown in Figure 5, compared with the vehicle control (*p* < 0.05 or *p* < 0.01), the membrane content of Rasa3 increased significantly, while that of cytosol decreased significantly after ADP stimulation, which indicated a membrane translocation of Rasa3. Different concentrations of BBR (Figures 5A,B) or M2 (Figures 5C,D) significantly inhibited the translocation of Rasa3 from the cytoplasm to membrane in the platelets upon ADP stimulation (*p* < 0.05 or *p* < 0.01 vs ADP treatment group). The inhibitory effect of BBR on Rasa3 membrane translocation was stronger than that of M2 when administered at the same concentration (*p* < 0.05) (Figures 5E,F). Actually, 0.5 μM of BBR showed an inhibitory effect similar to that of 100 nM of Wtm, which was used as a positive control in this experiment (Figures 5E,F).

### BBR and M2 Are Isoform-Selective Class I PI3Kβ Inhibitors

**FIGURE 6:**
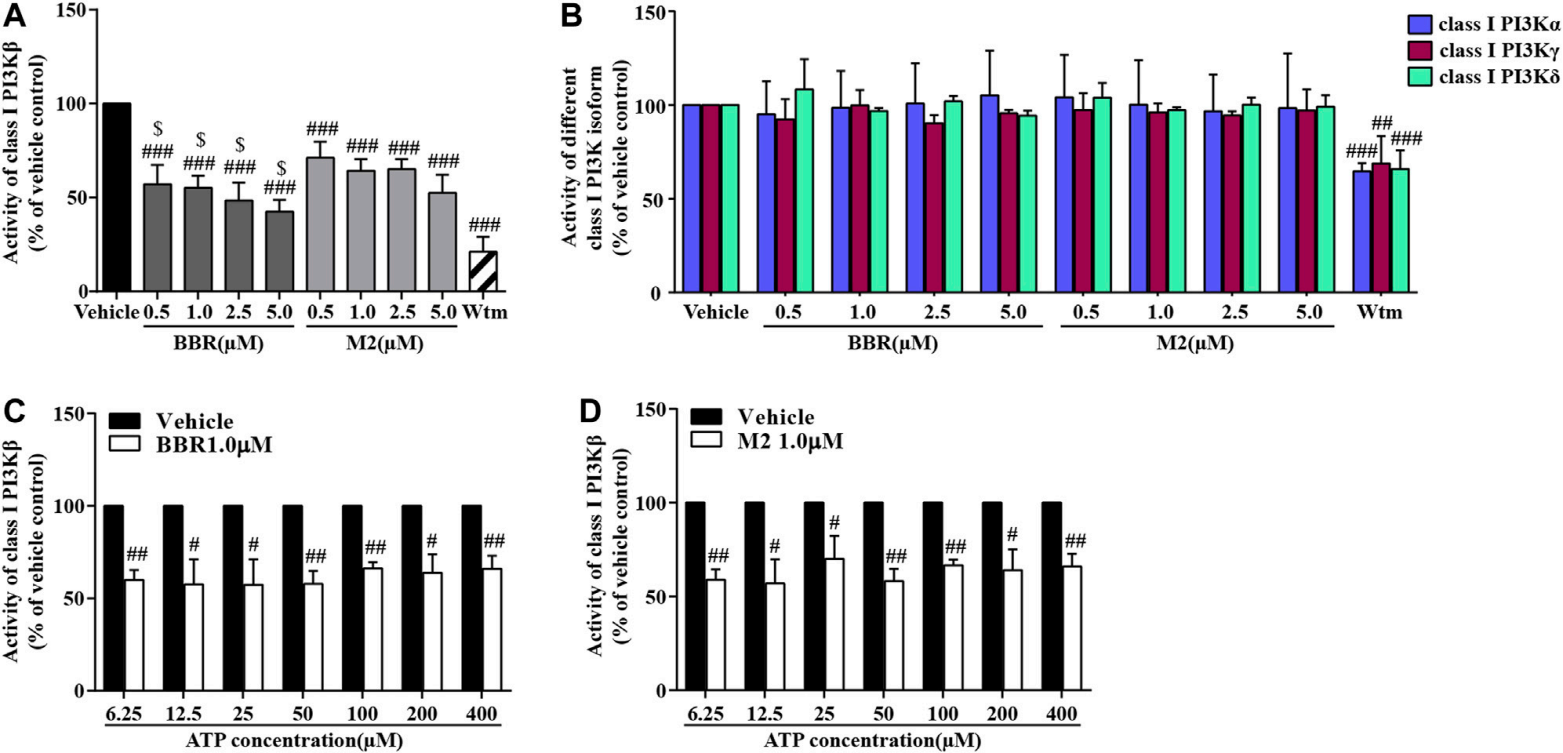
Effects of BBR and M2 on class I PI3K activities. **(A)** After treatment with the vehicle control (0.1% DMSO), BBR, M2, or Wtm (100 nM) for 20 min, the activities of class I PI3Kβ were determined. **(B)** After treatment, the activities of class I PI3Kα, γ, and δ were determined. **(C)** After vehicle or BBR treatment, a class I PI3Kβ activity assay was performed with different concentrations of ATP for 1 h. **(D)** After the vehicle control or M2 treatment, the class I PI3Kβ activity assay was performed with different concentrations of ATP for 1 h. Data are expressed as mean ± SD of three–five independent experiments. Statistically significant differences compared with the vehicle control group are indicated by ^#^ *p* < 0.05, ^##^ *p* < 0.01, and ^###^ *p* < 0.001, and statistically significant differences between the BBR and M2 groups at the same concentration are indicated by ^$^ *p* < 0.05, ^$$^ *p* < 0.01.

The ADP-Glo lipid kinase systems were used to detect the influences of BBR and M2 on the activities of different class I PI3K isoforms. As shown in Figure 6A, both BBR and M2 significantly inhibited class I PI3Kβ activity (*p* < 0.001 vs vehicle control), and BBR had a stronger inhibitory effect than M2 when administered at the same concentration (*p* < 0.05 or *p* < 0.01). As a positive control, Wtm also potently inhibited class I PI3Kβ activity (*p* < 0.001 vs vehicle control) (Figure 6A).

Interestingly, neither BBR nor M2 inhibited the activities of class I PI3Kα, PI3Kγ, or PI3Kδ (Figure 6B), which were greatly inhibited by Wtm (*p* < 0.01 or *p* < 0.001 vs vehicle control). These results indicate that BBR and M2 are isoform-selective class I PI3Kβ inhibitors.

The class I PI3Kβ protein contains an active site and an ATP catalytic site (Zhang et al., 2011). To explore the mechanisms of BBR and M2 to inhibit class I PI3Kβ, we observed whether or not their inhibitory effects were competitively inhibited by increasing ATP concentrations. As shown in Figures 6C,D, the inhibitory effects of BBR and M2 on class I PI3Kβ activity were not influenced when ATP concentration increased from 6.25 to 400 μM (*p* < 0.05 or *p* < 0.01 vs vehicle control).

**FIGURE 7:**
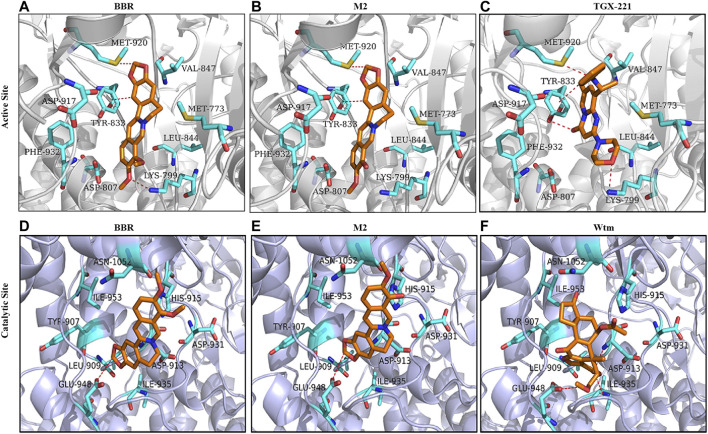
Molecular docking. Molecular docking was performed to study the 3D binding modes of BBR and M2 with the active site **(A-B)** and the catalytic site **(D-E)** of class I PI3K p110β. The binding modes of TGX221 with the active site **(C)** and Wtm with the catalytic site **(F)** are also presented. All studied compounds are in orange, the surrounding residues in the binding pockets are in cyan, and the backbone of the receptor is depicted as grey or lavender cartoon.

Then, docking simulation studies were carried out to investigate the binding modes of BBR and M2 with PI3K p110β. As known inhibitors of class I PI3Kβ, TGX211 and Wtm were used as positive controls for the active site (Marshall et al., 2015) and catalytic site (Yano et al., 1993) binding, respectively. The docking scores of BBR, M2, and TGX221 for the active site of PI3K p110β are shown in Table 1, which are −7.24, −6.93, and −7.88 kcal/mol, respectively. The binding mode of BBR with the active site of PI3K p110β is illustrated in Figure 7A. The oxygen atom of the methoxy radical of BBR, regarded as a hydrogen bond acceptor, forms a hydrogen bond with the nitrogen atom of the amino group of Lys799 in the active site of PI3K p110β. The carbon atom of the dioxolane ring of BBR, regarded as a hydrogen-bond donor, forms a hydrogen bond with the sulfur atom of the thioether group of Met920 in the active site of PI3K p110β. The carbon atom of isoquinoline of BBR forms H-π conjugate with the benzene ring of Tyr833 in the active site of PI3K p110β. The binding mode of M2 with the active site of PI3K p110β is illustrated in Figure 7B. The carbon atom of the dioxolane ring of M2, regarded as a hydrogen-bond donor, forms a hydrogen bond with the sulfur atom of the thioether group of Met920 in the active site of PI3K p110β. The carbon atom of isoquinoline of M2 forms H-π conjugate with the benzene ring of Tyr833 in the active site of PI3K p110β. As a positive control, the binding mode of TGX211 with the active site of PI3K p110β is illustrated in Figure 7C. The oxygen atom of the six-membered heterocycles of TGX211, regarded as a hydrogen-bond acceptor, forms a hydrogen bond with the nitrogen atom of the amino group of Lys799 in the active site of PI3K p110β. The oxygen atom of carbonyl of TGX211, regarded as a hydrogen-bond acceptor, forms a hydrogen bond with the oxygen atom of the phenolic hydroxyl group of Tyr833 in the active site of PI3K p110β. The nitrogen atom of TGX211, regarded as a hydrogen-bond donor, forms a hydrogen bond with the sulfur atom of the thioether group of Met920 in the active site of PI3K p110β. The carbon atom TGX211 forms a H-π conjugate with the benzene ring of Tyr833 in the active site of PI3K p110β.

**TABLE 1:** Docking scores of different ligands binding with PI3K p110β.

Ligand	Receptor	Docking score (kcal/mol)
BBR	PI3K p110β active site	−7.24
M2	PI3K p110β active site	−6.93
TGX211	PI3K p110β active site	−7.88
BBR	PI3K p110β catalytic site	−5.42
M2	PI3K p110β catalytic site	−5.14
Wtm	PI3K p110β catalytic site	−6.12

BBR, berberine; M2, berberrubine; Wtm, wortmannin.

The docking scores of BBR, M2, and Wtm for the catalytic site of PI3K p110β are shown in Table 1, which are −5.42, −5.14, and −6.12 kcal/mol, respectively. The binding mode of BBR with the catalytic site of PI3K p110β is illustrated in Figure 7D. The carbon atom of the dioxolane ring of BBR, regarded as a hydrogen-bond donor, forms a hydrogen bond with the oxygen atom of the carboxyl group of Glu948 in the catalytic site of PI3K p110β. The binding mode of M2 with the catalytic site of PI3K p110β is illustrated in Figure 7E. Similar to BBR, the carbon atom of the dioxolane ring of M2, regarded as a hydrogen-bond donor, forms a hydrogen bond with the oxygen atom of the carboxyl group of Glu948 in the catalytic site of PI3K p110β. The binding mode of Wtm with the catalytic site of PI3K p110β is illustrated in Figure 7F. The oxygen atom of carbonyl of Wtm, regarded as a hydrogen-bond acceptor, forms hydrogen bonds with the nitrogen atoms of the backbone of Asp913 and Phe939 in the catalytic site of PI3K p110β. The carbon atom of Wtm, regarded as a hydrogen-bond donor, forms a hydrogen bond with the oxygen atom of the carboxyl group of Glu948 in the catalytic site of PI3K p110β. Taken together, the computational results indicate that BBR and M2 are more likely to bind to the PI3K p110β active site rather than the catalytic site, which is in agreement with the results of the ATP competition experiment (Figures 6C,D).

### BBR Inhibits Platelet Activation Through Suppressing the PI3K/Akt Pathway and Rap1 Activation *ex vivo*

**FIGURE 8:**
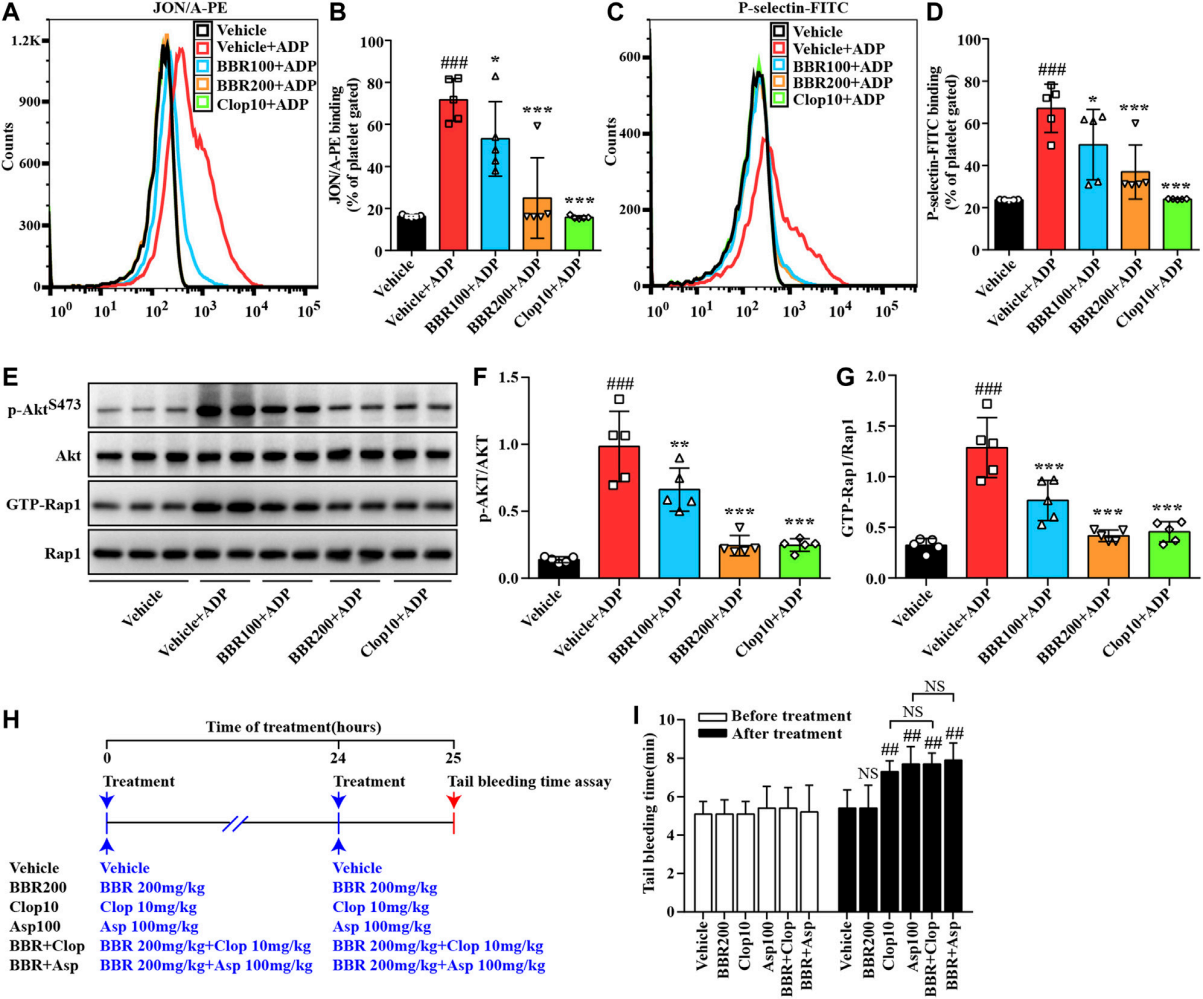
Effects of BBR on platelet activation *ex vivo* and the tail bleeding time in mice. Twenty-five C57BL/6N mice were divided into five groups, and each group received the treatment as indicated. The WPs were prepared and treated with the vehicle control (0.1% DMSO) or ADP for 10 min, and the percent of JON/A-PE–positive platelets **(A-B)** and P-selectin-FITC–positive platelets **(C-D)** were determined by flow cytometry. Proteins were extracted for pull-down and Western blot analysis of p-Akt^S473^ and GTP-Rap1 levels **(E-G)**, which were normalized to Akt and Rap1, respectively, and plotted as indicated. Representative images of flow cytometry **(A, C)** and protein blots **(E)** are shown. For quantitative data **(B, D, F, G)**, values are expressed as mean ± SD of five mice in each group. Statistically significant differences compared with the vehicle control group are indicated by ^###^ *p* < 0.001, and statistically significant differences compared with the vehicle control + ADP group are indicated by *p < 0.05, **p < 0.01, and ***p < 0.001. In a parallel experiment, 30 mice were divided into six groups and treated as indicated **(H)**. Before and after the treatment, the tail bleeding time was determined **(I)**. Values are expressed as mean ± SD of five mice in each group, and statistically significant differences compared with the vehicle control group are indicated by ^##^ *p* < 0.01.

To explore the suppressive effects of BBR on platelet activation *ex vivo*, the mice were orally administrated with 100 mg/kg or 200 mg/kg BBR for 14 days. After treatment, WPs were prepared and stimulated with 10 μM of ADP. Compared with the ADP-stimulated group (*p* < 0.05 or *p* < 0.001), 100 mg/kg and 200 mg/kg of BBR significantly reduced the process of positive platelets with PE-conjugated JON/A antibody binding (Figures 8A,B) or FITC-conjugated P-selectin antibody binding (Figures 8C,D) in a dose-dependent manner.

Compared with the ADP-stimulated group (*p* < 0.01 or *p* < 0.001), the oral treatment of BBR also inhibited the phosphorylation of Akt and suppressed Rap1 activation (Figures 8E–G) dose-dependently. The *ex vivo* inhibitory effects of BBR at 200 mg/kg on platelet activation (Figures 8A–D), Akt phosphorylation (Figures 8E,F), and Rap1 activation (Figures 8E,G) were similar to those of Clop at 10 mg/kg (Clop10), which was used as a positive control in this animal experiment.

### BBR Does Not Prolong Tail Bleeding Time

As shown in Figure 8I, there was no statistically significant difference observed among the studied groups before treatment. After oral administration with 100 mg/kg of Asp or 10 mg/kg of Clop, the tail bleeding time in the mice was prolonged significantly as compared to the vehicle control group (*p* < 0.01). For comparison purposes, 200 mg/kg of BBR had no influence on the tail bleeding time in the mice. Moreover, when BBR was used in combination with Asp or Clop, it did not further prolong the tail bleeding time in the mice (Figure 8I).

### BBR Suppresses Carrageenan-Induced Thrombosis

**FIGURE 9:**
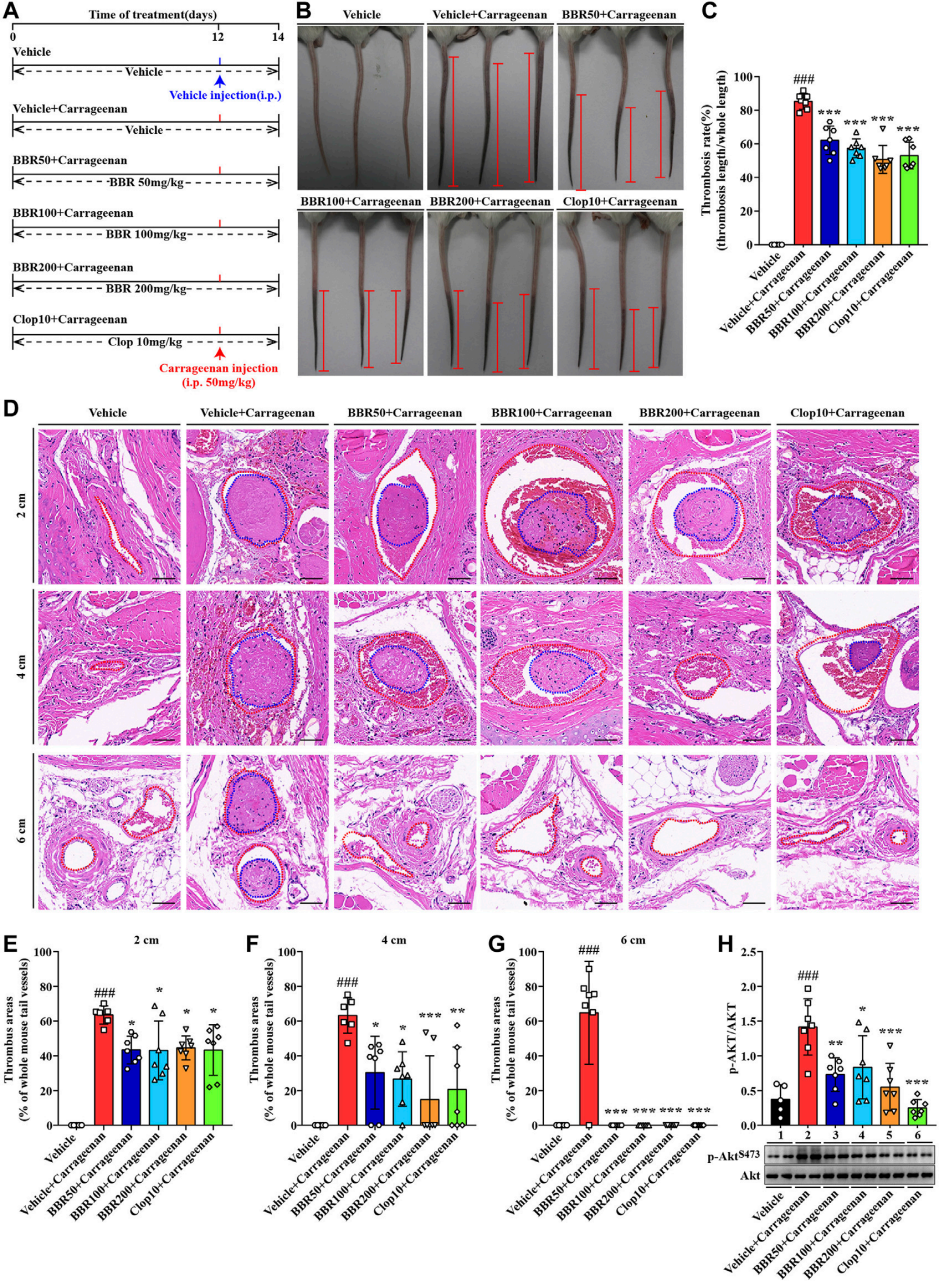
Effects of BBR on carrageenan-induced thrombosis and Akt phosphorylation in mice. The experimental design **(A)**. Two days after carrageenan injection, the tails of the mice were photographed, and the representative images are presented **(B)**, in which the black parts in the tails of the mice are thrombus and the red segments indicate the length of thrombus. The thrombosis rate was calculated and plotted as indicated **(C)**. The mice were sacrificed, and their tails were subjected to paraffin sections at 2, 4, and 6 cm away from the tail tips. The sections were stained by H&E, and the typical images are presented **(D)** (×200, scale bar = 50 µm). The red dotted lines represent tail vessels and the blue dotted lines represent thrombi. Thrombus areas at 2, 4, and 6 cm away from the tail tips were quantified and are presented as the percentages of the whole tail vessels of the mice **(E-G)**. The WPs were used for protein extraction and Western blot analysis of the p-AKT^S473^ level **(H)**, which was normalized to Akt and plotted as indicated. Data are represented as mean ± SD of five or seven mice in each group. Statistically significant differences compared with the vehicle control group are indicated by ^###^ *p* < 0.001, and statistically significant differences compared with the vehicle + carrageenan group are indicated by *p < 0.05, **p < 0.01, ***p < 0.001.

A carrageenan-induced thrombosis model was used to investigate the inhibitory effects of BBR on thrombus formation. The animal experimental scheme is shown in Figure 9A. After i.p. injection, carrageenan induced obvious thrombus formation in the tails of the mice (Figure 9B) with an average thrombosis rate of more than 80% (*p* < 0.001 vs vehicle control) (Figure 9C). The oral administration of BBR at 50, 100, or 200 mg/kg effectively inhibited carrageenan-induced thrombosis (Figure 9B) and reduced the thrombosis rate in the tails of mice dose-dependently (*p* < 0.001 vs vehicle + carrageenan group) (Figure 9C). Clop at 10 mg/kg inhibited thrombus formation and reduced the thrombosis rate potently (*p* < 0.001 vs vehicle + carrageenan group), and the effects were similar to those of BBR at 200 mg/kg (Figures 9B,C).

The tails of the mice were subjected to pathological examination, and the results showed that in the vehicle + carrageenan group, the tail vessel lumen was almost completely occupied by thrombus (Figure 9D), and the percentages of the thrombus area exceeded 60% at 2, 4, and 6 cm from the tail tips (*p* < 0.001 vs vehicle control) (Figures 9E–G). BBR at different doses greatly reduced the thrombus area at different locations in the tails of the mice (Figures 9D–G). For instance, at 4-cm position from the tail tips, the thrombus area was reduced by about 29.6, 55.9, and 75.5% after receiving a low, middle, or high dose of BBR treatment, respectively (*p* < 0.05 or *p* < 0.001 vs vehicle + carrageenan group) (Figure 9F). Furthermore, there was no thrombus observed at the 6-cm position after different doses of BBR treatment (*p* < 0.001 vs vehicle + carrageenan group) (Figures 9D,G). As a positive control, Clop reduced the thrombus area to an extent similar to that of BBR at 200 mg/kg (Figures 9D–G).

Accompanied by thrombus formation, carrageenan significantly activated the PI3K/Akt signaling pathway in the platelets, as indicated by a significant increase in the p-Akt level after injection (*p* < 0.001 vs vehicle control) (Figure 9H). The oral administration of BBR (50, 100, and 200 mg/kg) or Clop significantly inhibited the phosphorylation of Akt in platelets (*p* < 0.05, *p* < 0.01, or *p* < 0.001 vs vehicle + carrageenan group) (Figure 9H).

## Discussion

In this study, we report for the first time that BBR and its main metabolite M2 inhibit platelet activation through suppressing the class I PI3Kβ/Rasa3/Rap1 pathway.

The active forms of integrin αIIbβ3 and P-selectin on the platelet surface are two biomarkers of platelet activation (Bath et al., 2018; Huang et al., 2019). In our experiments, the direct evidence that BBR and M2 inhibit platelet activation was seen in the downregulation of their expression on the platelet surface upon ADP stimulation, both *in vitro* and *ex vivo*. In addition, upon stimulation with ADP or other agonists, the conformation of integrin αIIbβ3 changes from an inactive form to an active form, which lets it bind to fibrinogen in the blood and promote platelet aggregation and thrombosis. Therefore, the reduction of the binding ability of the platelets to fibrinogen is another evidence to support the inhibitory effects of BBR and M2 on platelet activation.

We found that BBR and M2 inhibited the binding of platelets to fibrinogen but did not affect the binding of purified αIIbβ3 to fibrinogen, which suggests that they might inhibit platelet activation through regulating certain intracellular signaling pathways rather than directly inhibiting integrin αIIbβ3. This inference was verified by experimental results that BBR and M2 blocked PI3K/Akt and Rap1 activation induced by ADP.

The PI3K/Akt signaling pathway is a common pathway for platelet activation and aggregation induced by a variety of agonists such as ADP, collagen, and arachidonic acid (Chu et al., 1989; Valet et al., 2016). There are many isoforms of PI3Ks which include class I PI3K (α, β, γ, δ), class II PI3Kα and β, and class III PI3K (Valet et al., 2016). Among these isoforms, only class I PI3K could catalyze the conversion of PIP2 to phosphatidylinositol-3,4,5-trisphosphate (PIP3), which is a second messenger and participated in platelet activation through binding with several target proteins (Valet et al., 2016). In this study, we found that BBR and M2 significantly inhibited the phosphorylation of Akt, both *in vitro* and *ex vivo*, which directly represented their suppressive activities on PI3K signaling. The kinase assay proved that BBR and M2 selectively inhibited class I PI3Kβ, and this was the most interesting and surprising finding in our study. In addition, the ATP competition experiment and computational simulation suggested that the target of BBR and M2 on class I PI3Kβ was probably its active site on the subunit of p100.

**FIGURE 10:**
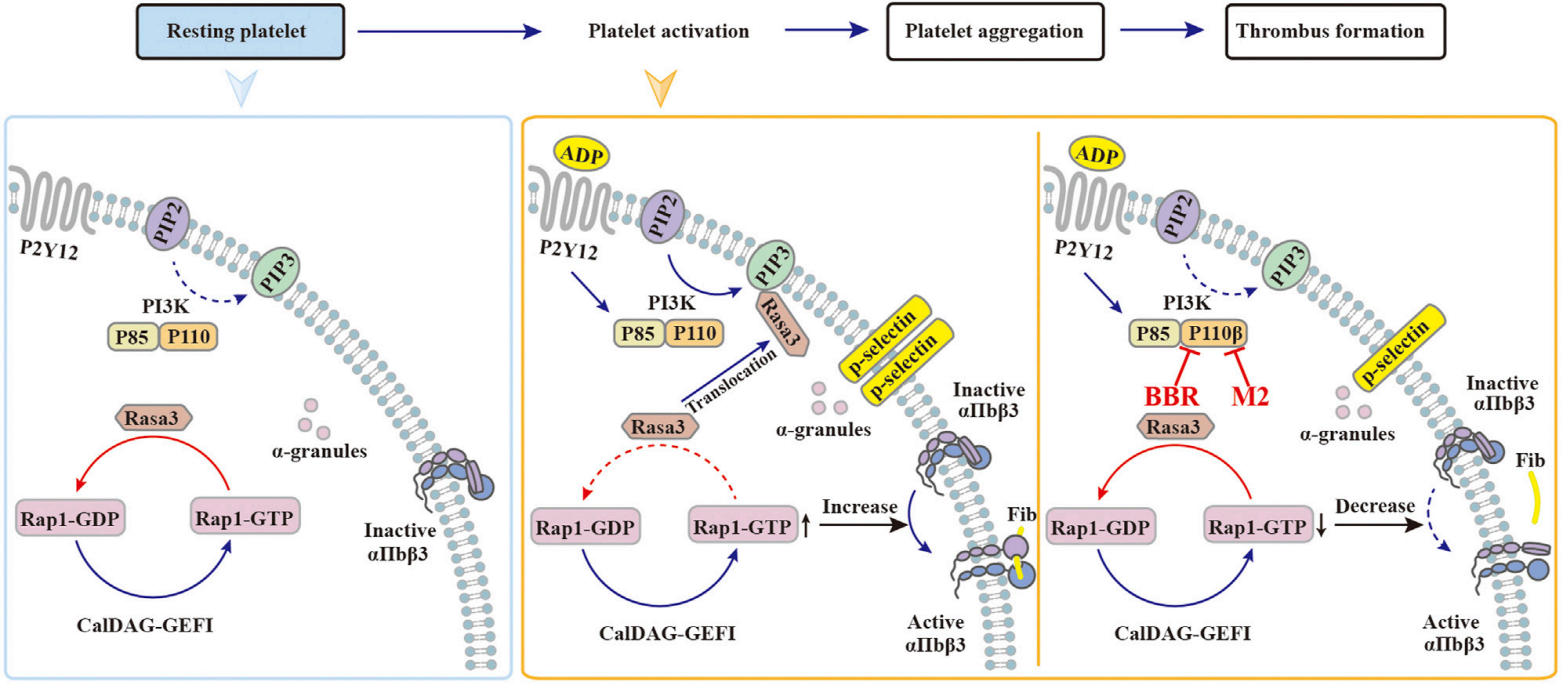
Possible mechanisms of BBR to inhibit platelet activation. **(1)** In resting platelets, the majority of Rasa3 are located in the cytoplasm, while PI3K and CalDAG-GEFI jointly maintain the balance between GDP-Rap1 and GTP-Rap1. **(2)** When stimulated by agonists such as ADP, the PI3K signaling pathway is activated, and Rasa3 will translocate from the cytoplasm to the cell membrane, which is recruited by PIP3. As a result, the balance between GDP-Rap1 and GTP-Rap1 is broken, which causes the activation of integrin αIIbβ3 and the platelets. **(3)** When the platelets are pretreated with BBR or M2, the catalytic activity of class I PI3K p110β is inhibited. Rasa3 will not translocate to the cell membrane and will remain in the cytoplasm, which will help to maintain the balance between GDP-Rap1 and GTP-Rap1. In this manner, the integrin αIIbβ3 keeps an inactive form, and the platelet activation is inhibited by BBR or M2.

Upon activation, class I PI3Kβ plays a significant role in promoting Rasa3 membrane translocation, Rap1 activation, and the subsequent activation and adhesion of integrin αIIbβ3 (Su et al., 2016; Valet et al., 2016; Jackson et al., 2005), which will facilitate thrombus formation (Figure 10). In resting platelets, Rasa3 and the factor CalDAG-GEFI jointly balance the level of GTP-Rap1 and GDP-Rap1 (Zhu et al., 2017). When the platelets are stimulated with agonists, Rasa3 is recruited from the cytoplasm to the cell membrane by PIP3, the dynamic balance of GTP-Rap1 and GDP-Rap1 is broken, and the level of GTP-Rap1 increases, which binds and activates integrin αIIbβ3 and finally activates platelets (Figure 10). Through selective inhibition of class I PI3Kβ, BBR and M2 can block Rasa3 membrane translocation, Rap1 activation, and integrin αIIbβ3 activation. Our results suggest that BBR and M2 inhibit platelet activation through suppressing the class I PI3Kβ/Rasa3/Rap1 pathway, which is summarized in Figure 10.

BBR is a multi-target drug, and it is proper to infer that it may suppress platelet activation through multiple mechanisms. For example, in addition to Rasa3 and Rap1, Akt (including AKT1, AKT2, and AKT3 isoforms) as a downstream molecule of PI3K may also participate in the antiplatelet activities of BBR. As previous reports have proved the deletion of the AKT1 (Chen et al., 2004) or AKT2 (Woulfe et al., 2004) gene could inhibit platelet activation and aggregation upon stimulation of various agonists *in vitro*, and the deletion of the Akt2 (Woulfe et al., 2004) or Akt3 (O'Brien et al., 2011) gene could inhibit (ferric chloride) FeCl_3_-induced carotid artery thrombus formation *in vivo*.

In addition, according to a recent report, regulation of mitochondrial function may be a promising strategy to inhibit platelet activation (Fuentes et al., 2019). BBR is reported to have an influence on mitochondrial function (Kumar et al., 2015); therefore, its antiplatelet activities may be associated with the modulation of mitochondrial function, which merits further investigation.

We noticed that the inhibitory effects of BBR on platelet activation and the class I PI3Kβ/Rasa3/Rap1 pathway are stronger than those of M2, its main phase I metabolite. These findings agree with our previous studies, in which M2 had shown moderate BBR-like biological activities, such as low-density lipoprotein (LDLR) upregulation, insulin receptor (InsR) upregulation, and AMP-activated protein kinase (AMPK) activation (Li et al., 2010; Li et al., 2011; Wang et al., 2012). The activities of M2 on these molecular targets are approximately 59–68% of those of BBR (Li et al., 2010; Li et al., 2011; Wang et al., 2012). Due to the inner biological activities of M2, *in vivo* antiplatelet efficacies of BBR may be attributable to a combination of itself and M2, which needs further studying. In addition, whether or not other phase I metabolites of BBR have similar antiplatelet activities is unknown and needs further investigation. The clarification of the structure–activity relationship of BBR metabolites on inhibiting platelet activation is of scientific and practical significance, and relevant experiments are now ongoing in our laboratory.

The antiplatelet efficacy of BBR is translated into a suppressive activity on thrombus formation *in vivo*. In this study, we show that BBR greatly inhibits carrageenan-induced thrombosis in the tails of the mice after oral administration. Our findings are in agreement with previous reports, in which BBR suppressed thrombus formation in the inferior vena cava (Wang et al., 2018), cerebral artery (Wu and Liu, 1995), and lungs (Li et al., 1994). As a multi-target drug, BBR may suppress thrombus formation through multiple mechanisms in addition to the antiplatelet effect. For example, BBR is reported to have anticoagulant activities (Wang et al., 2017; Wang et al., 2018), and it also has protective activities on the vascular endothelium (Guo et al., 2016) and beneficial effects on hemodynamics (Xie et al., 2011). These effects of BBR may also contribute to its antithrombotic activity and support its future clinical application in the prevention or treatment of thrombotic diseases or cardiovascular/cerebrovascular events.

One of the major advantages of BBR is its good safety (Yao et al., 2015). In this study, BBR alone had no influence on the tail bleeding time in the mice, and when used in combination with Clop or Asp, it did not further prolong the tail bleeding time. This phenomenon may be explained as the following: first, the blood concentration of BBR is relatively low after oral administration and is not sufficient to cause bleeding, and second, rather than potently modulating a single target, such as other commonly used antiplatelet drugs, BBR has pleiotropic effects on a variety of targets, which will minimize its adverse effects (Kong et al., 2020). We consider that at this point, BBR may have an advantage over other antiplatelet drugs, and it is suitable for combination usage with other antiplatelet drugs in future clinical applications.

In conclusion, our studies reveal that the natural product BBR and its main metabolite M2 inhibit platelet activation through suppressing class I PI3Kβ, Rasa3 membrane translocation, and then Rap1 activation and that the antiplatelet activities of BBR are effectively converted to an antithrombotic efficacy *in vivo* without increasing the bleeding risk. These properties suggest that BBR may be a promising antiplatelet drug that can be used in the prevention or treatment of thrombotic diseases or cardiovascular/cerebrovascular events.

## Data Availability

The original contributions presented in the study are included in the article/Supplementary Material; further inquiries can be directed to the corresponding authors.
