# Supplementary material for: Berberine and Its Main Metabolite Berberrubine Inhibit Platelet Activation Through Suppressing the Class I PI3Kβ/Rasa3/Rap1 Pathway
Source: Front Pharmacol. 2021 Oct 8;12:734603. doi: 10.3389/fphar.2021.734603 (PMC8531212; doi:10.3389/fphar.2021.734603)
Supplement: Supplementary file 1 [file Table1.docx]

Supplementary Material

**Berberine and its main metabolite berberrubine inhibit platelet activation through suppressing the class I PI3Kβ/Rasa3/Rap1 pathway**

**Can Wang^1,2^, Yangyang Cheng^3^, Yuanhui Zhang^3^, Hongtao Jin^4^, Zengyan Zuo^3^, Aiping Wang^4^, Jianmei Huang^1^*, Jiandong Jiang^2,3^*, and Weijia Kong^3^***

^1^School of Chinese Materia Medica, Beijing University of Chinese Medicine, Beijing, China

^2^State Key Laboratory of Bioactive Substance and Function of Natural Medicines, Institute of Materia Medica, Chinese Academy of Medical Sciences and Peking Union Medical College, Beijing, China

^3^Department of Virology & NHC Key Laboratory of Biotechnology of Antibiotics, Institute of Medicinal Biotechnology, Chinese Academy of Medical Sciences and Peking Union Medical College, Beijing, China.

^4^New Drug Safety Evaluation Center, Institute of Materia Medica, Chinese Academy of Medical Sciences and Peking Union Medical College, Beijing, China.

*** Correspondence:**Jianmei Huang

hjm70@139.com

Jian-Dong Jiang

jiang.jdong@163.com

Wei-Jia Kong

kongweijia@imb.pumc.edu.cn


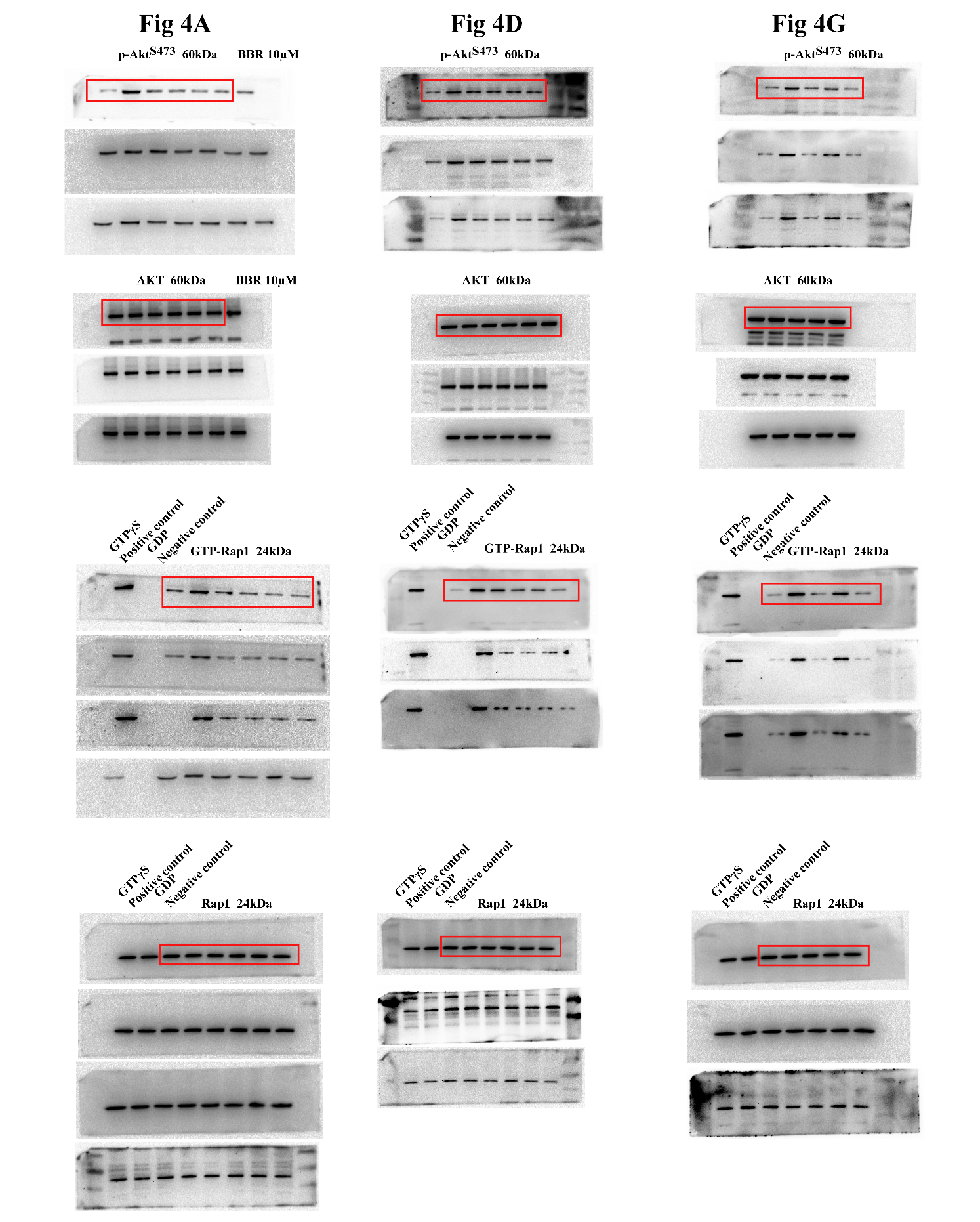


**Supplementary Figure 1.** **Original scans of western blot in Figure 4.**

**
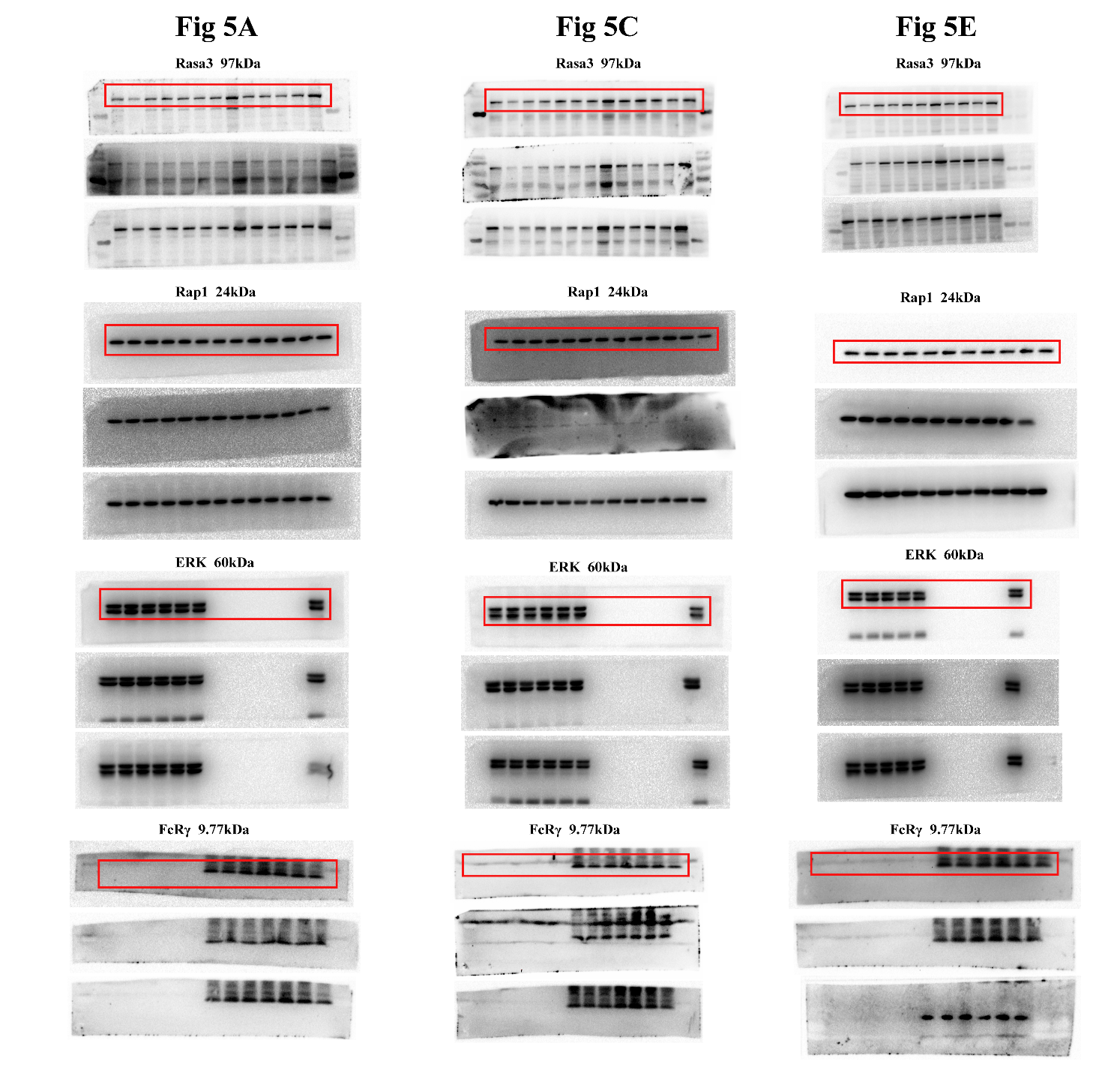
**

**Supplementary Figure 2. Original scans of western blot in Figure 5.**

**
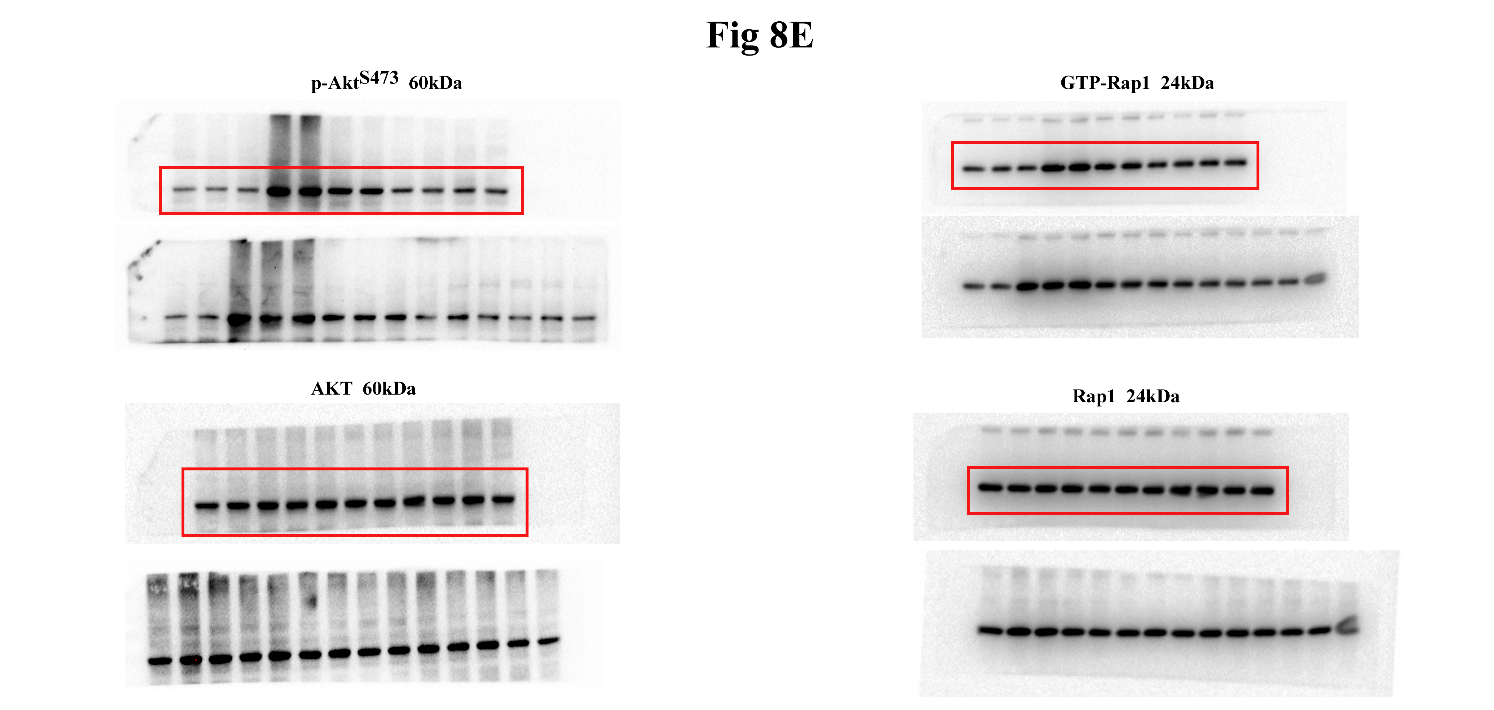
**

**Supplementary Figure 3. Original scans of western blot in Figure 8.**

**
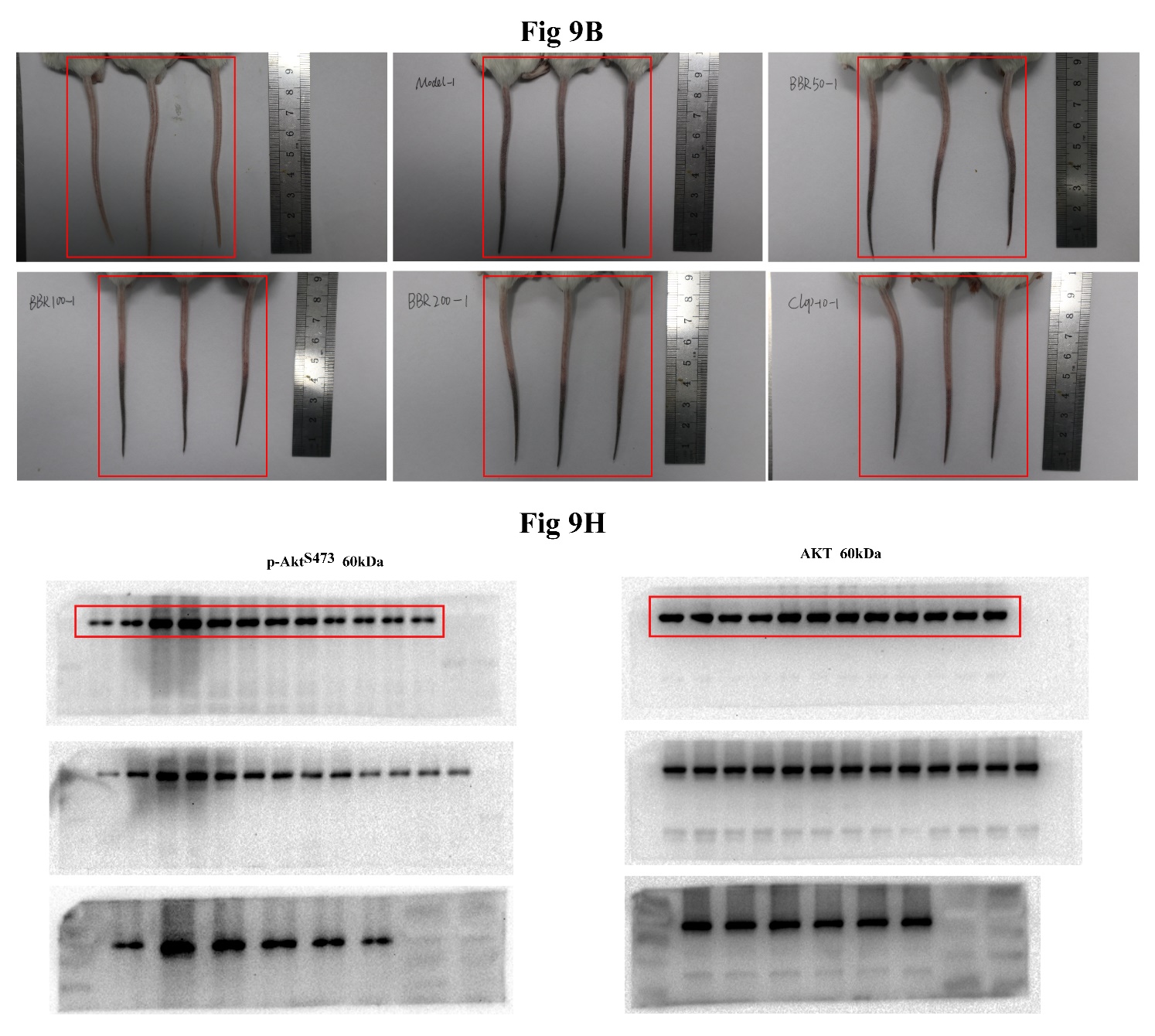
**

**Supplementary Figure 4. Original images of mouse tail thrombosis and original scans of western blot in Figure 9.**
